# Data mobilisation at the Fund of Invertebrates of the State Museum of Natural History of the NAS of Ukraine

**DOI:** 10.3897/BDJ.12.e131188

**Published:** 2024-08-20

**Authors:** Andriy Novikov, Volodymyr Rizun, Andrii Susulovsky, Habriel Hushtan, Kateryna Hushtan, Oleksandr Kuzyarin, Anastasiia Savytska, Viktor Nachychko, Solomia Susulovska, Dmytro Leleka

**Affiliations:** 1 State Museum of Natural History of the NAS of Ukraine, Lviv, Ukraine State Museum of Natural History of the NAS of Ukraine Lviv Ukraine; 2 Separate Structural Department “Lviv Professional College of the Lviv National Environmental University”, Lviv, Ukraine Separate Structural Department “Lviv Professional College of the Lviv National Environmental University” Lviv Ukraine; 3 Ivan Franko National University of Lviv, Lviv, Ukraine Ivan Franko National University of Lviv Lviv Ukraine; 4 Institute of Ecology of the Carpathians of the NAS of Ukraine, Lviv, Ukraine Institute of Ecology of the Carpathians of the NAS of Ukraine Lviv Ukraine

**Keywords:** Arthropoda, Oribatida, Coleoptera, Lepidoptera, Nematoda, invertebrates, museum collection, data mobilisation, Ukraine

## Abstract

**Background:**

The described dataset contains occurrence records of invertebrate specimens deposited at the State Museum of Natural History of the NAS of Ukraine, Lviv, Ukraine (SMNH NASU). It combines diverse taxonomic groups, mostly belonging to the class Insecta of the phylum Arthropoda, that were selected as prioritised for digitisation in war conditions. Selected specimens were ascertained as those being the most vulnerable to hostilities and requiring virtual preservation. Such virtual preservation is essential in the war realities as collection can be lost or damaged at any moment, resulting in a significant retrospective biodiversity data gap. At the same time, collection virtualisation and its deposition on the internet grant remote access to scientists who cannot visit it in person due to the war. Moreover, we believe that the mobilisation of the data from the Ukrainian collections and their publication online are essential for the integration of Ukrainian research facilities into a global scientific biodiversity pool.

**New information:**

A total of 3,660 occurrence records mobilised in 2023-2024 from the collection of invertebrates of the SMNH NASU, were published. This dynamic dataset will be continually supplied by new records during further digitisation work.

## Introduction

The digitisation of natural history collections and mobilisation of biodiversity data are crucial tasks significantly influencing modern science, providing access to growing data arrays (Nelson et al. 2015, Wetzel et al. 2018, Ball-Damerow et al. 2019). It is realised in multiple ways, including numerous local initiatives (e.g. Vattakaven et al. (2016), Grattarola et al. (2019), Guzmán-Jacob et al. (2021), Novotný et al. (2022), Gyeltshen and Pem (2022)), citizen-science platforms like iNaturalist (2024), Observation International (2024) and eBird (2024), as well as global aggregators like GBIF (2024a) or iDigBio (2024). Mobilised data contributes to an extendable number of biogeographical, taxonomical, phylogenetical, ecological and other biodiversity-related investigations (Causey et al. 2004, Holmes et al. 2016, Nelson and Ellis 2018, Shultz et al. 2020, Heberling et al. 2021). In light of the continuing hostilities in Ukraine, the mobilisation of biodiversity data and, particularly, digitisation of nature history collections, acquires a new sense.

SMNH NASU, being located in Lviv, far from the war line, is, however, suffering from indirect impacts such as lack of financial support due to govermental budget transfer to the prioritised defence policy and damage to hosted collections as a result of blackouts and lack of heating. The scientific fund of invertebrates of the SMNH NASU comprises several independent collections each curated by their custodians and curators. There are collections of extant and fossil insects, molluscs, microscopic slides of the soil invertebrates (nematodes, flatworms, springtails, protura, oribatid and mesostigmatic mites etc.) and a few memorial collections. In general, the fund is subdivided into the principal and supplementary subfunds. The principal subfund of invertebrates includes ca. 188,000 storage units and the supplementary subfund has over 13,000 storage units. There are represented specimens collected since the end of 19^th^ and the beginning of the 20^th^ centuries by many famous regional naturalists, including Kazimierz Smulikowski, Marian and Jarosław Łomnicki, Maksymilian Nowicki, Michał Świątkiewicz, August Stöckl and Stanisław Kapuściński (Starzyk 2004).

**Figure 1.**
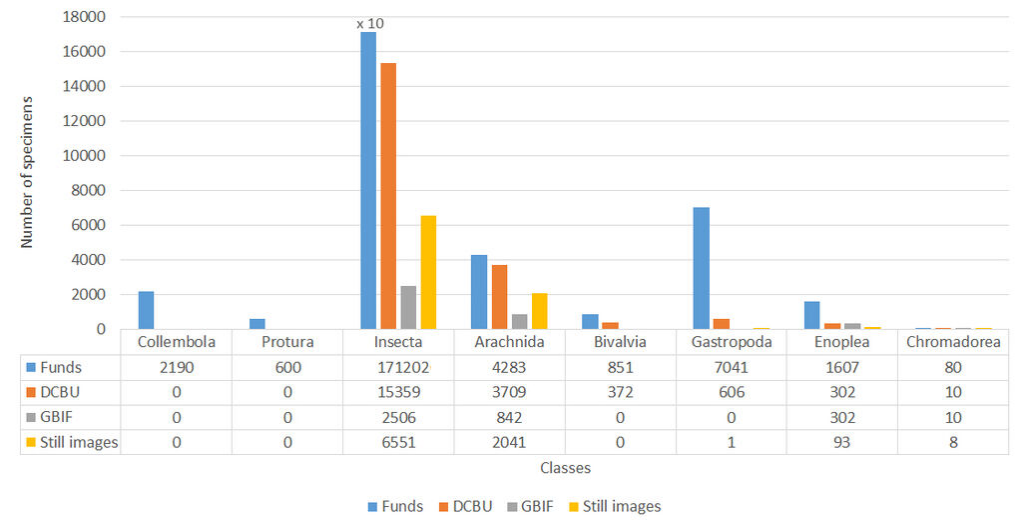
Digitisation progress of the invertebrates at the SMNH NASU.

The digitisation of the collections at the SMNH NASU started in the early 2000s when separated datasets were created locally by curators. In 2017, the Data Centre “Biodiversity of Ukraine” (DCBU) has been launched (State Museum of Natural History of the NAS of Ukraine 2024). Since then, it has served as the main entry point to host and operate with digitised materials (Rizun and Scherbachenko 2019, Rizun et al. 2020). By 2024, 20358 records about invertebrate specimens hosted at SMNH NASU, were deposited at the DCBU, including 19068 records of Arthropoda (Insecta - 15359, Arachnida - 3709), 978 records of Mollusca (Bivalvia - 372 and Gastropoda - 606) and 312 records of Nematoda (Enoplea - 302 and Chromadorea - 10). Thus, today, the DCBU contains data about 11% of the total number of invertebrate specimens hosted at SMNH NASU. Since late 2023, to provide wider access, the integration of mobilised data to the GBIF platform has begun. At the moment, data for only about 2% of the total number of invertebrate specimens hosted at SMNH NASU were published in GBIF and represented in the current dataset. Only 5% of the hosted specimens had images of different quality, which were captured at different times. Currently, many of these images are archived on the internal SMNH NASU servers and their publishing online through the DCBU and other platforms is still in progress. (Fig. 1).

## Project description

### Title

Digitisation of natural history collections damaged as a result of hostilities and related factors: development of protocols and implementation on the basis of the State Museum of Natural History of the National Academy of Sciences of Ukraine (Nr 2022.01/0013)

### Personnel

**Project PI**: Andriy Novikov (Dr., Senior Research Scientist, SMNH, Department of Biosystematics and Evolution, ORCID https://orcid.org/0000-0002-0112-5070).

**Core Team**: Habriel Hushtan (Dr., Research Scientist, SMNH, Department of Biosystematics and Evolution, ORCID https://orcid.org/0000-0001-6999-6043), Kateryna Hushtan (Dr., Research Scientist, SMNH, Department of Museum Informative Systems, ORCID https://orcid.org/0000-0002-5235-3233), Oleksandr Kuzyarin (Dr., Research Scientist, SMNH, Department of Museum Informative Systems, ORCID https://orcid.org/0000-0002-7728-3665), Bohdan Prots (Dr., Head of Department, SMNH, Department of Landscape and Biotic Diversity, ORCID https://orcid.org/0000-0002-0605-9527 - left the team in 2024), Volodymyr Rizun (Dr., Head of Department, SMNH, Department of Museum Informative Systems, ORCID https://orcid.org/0000-0002-1675-032X), Anastasiia Savytska (Dr., Research Scientist, SMNH, Department of Applied Museology, ORCID https://orcid.org/0000-0002-6255-8590), Andrij Susulovsky (Dr., Head of Department, SMNH, Department of Biosystematics and Evolution, ORCID https://orcid.org/0000-0002-4233-9825).

**Assistants**: Viktor Nachychko (Dr., Associate Professor, Ivan Franko National University of Lviv, Faculty of Biology, Department of Botany, ORCID https://orcid.org/0000-0001-6756-2823), Solomia Susulovska (Dr., Collection Keeper, Junior Research Scientist, Ivan Franko National University of Lviv, Faculty of Biology, Zoological Museum, ORCID https://orcid.org/0000-0001-7585-7584), Dmytro Leleka (PhD Student, Institute of Ecology of the Carpathians of the National Academy of Sciences of Ukraine, Department of Ecosystemology, ORCID https://orcid.org/0000-0002-0112-5070).

### Study area description

The study is focused on the invertebrate specimens collected mostly in the western part of Ukraine. However, the dataset also contains many occurrence records from other adjacent territories and occasional important records from remote areas such as Morocco, Kazakhstan, Kyrgyzstan, Sri Lanka and the Kuril Archipelago.

### Design description

As it was initially mentioned in our previous publication (Novikov et al. 2024), the project aims to: (a) develop digitisation protocols for the most valuable and vulnerable natural history collections; (b) mobilise and publish the data about such collections deposited at SMNH; and (c) digitise prioritised specimens deposited at SMNH, including those belonging to the herbarium collection and the collection of invertebrates. However, the digitisation workflow for invertebrate specimens differs from those for the herbarium material described by Novikov et al. (2024). In the case of digitisation of the herbarium material, there are two stages of capturing images - the first, preliminary, when the images of the herbarium labels are taken and the second, main, when the images of the entire herbarium sheets are taken. The data from the herbarium labels are manually transferred to the dataset and later verified for taxonomic consistency and other issues. Only after such verification, specimens meeting preselection and quality criteria, are digitised using a hi-res photo camera. In the case of invertebrates, the traditional digitisation workflow (Flemons and Berents 2012, Nelson et al. 2015, Blagoderov et al. 2017, Harris and Marsico 2017, Dupont et al. 2020, Nieva de la Hidalga et al. 2020) has been chosen - the specimens are preselected by curators, based on the preliminary outlined criteria and digitised. The data are later mobilised from the original images. Such a protocol simplifies the digitisation procedure and is excellent for routine digitisation of the entire collection. However, it loses in specimens preselection, resulting sometimes in the digitisation of less important specimens, misidentified specimens, mix of specimens from different regions and occasional records with low data quality (e.g. unknown collection date, uncertain locality etc.).

### Funding

The grant programme “Science for the Recovery of Ukraine in the War and Post-War Periods” (Nr 2022.01) of the National Research Foundation of Ukraine (NRFU).

## Sampling methods

### Sampling description

**Figure 2.**
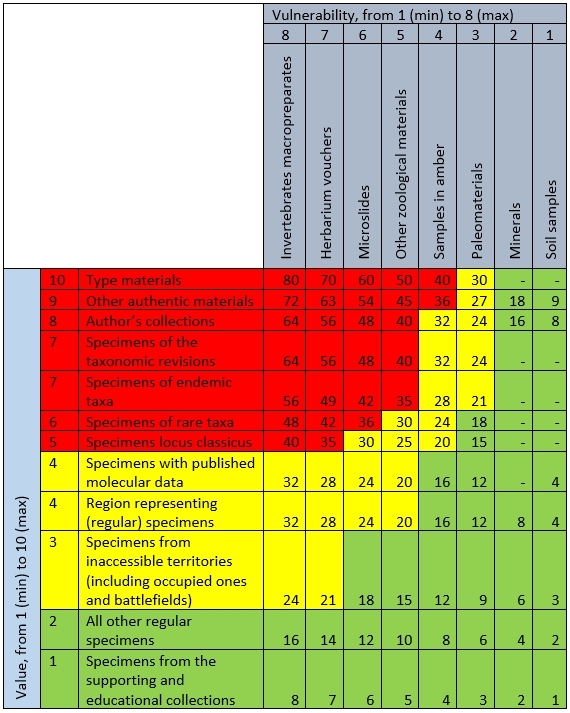
Simplified priority evaluation applied for the digitisation of the natural history collections at the SMNH NASU. Scores are calculated by multiplication of specimen value by its vulnerability. Three priority groups are ascertained - red (highest) with scores over 35; yellow (moderate) with scores between 20 and 35; and green (lowest) with scores under 20.

Similarly to the botanical fund (Novikov et al. 2024), three levels of priority for digitisation and data mobilisation were defined in other funds of SMNH NASU (Fig. 2). The first, red, group comprises the most valuable specimens that are, at the same time, the most vulnerable (e.g. can be easily and heavily damaged by moisture, fire, mould etc.). The second, yellow, group combines valuable specimens that are relatively resistant to damage and specimens that also can be easily damaged, but have moderate importance. The third, green, group includes specimens of regular species and specimens from supporting (e.g. loan and educational) collections that are either resistant to damage or have limited scientific value.

Although the mentioned priority classification (Fig. 2) is not constant, we believe that general logic of designation of the priority groups by multiplication of the specimen value by its vulnerability could be useful for other digitisation projects. In particular, such priority classification can serve as a good point to focus the digitisation in emergency situations. At the same time, the number of value and vulnerability levels, as well as their order, can be determined individually, depending on the collection peculiarities (e.g. presence of type material and preservation technique) and certain digitisation goals (e.g. in case of long-term flow digitisation, such priority groups can be neglected or bulked).

### Step description

1. To designate the digitisation priority, the working table with classes of value (from 1 to 10) and vulnerability (from 1 to 8) of the specimens has been created (Fig. 2). Each specimen was evaluated following these two principal criteria and received cumulative points. For example, if the specimen is a herbarium voucher (7^th^ level of vulnerability) representing the endemic taxon (7^th^ level of value), it received 49 (7 x 7) cumulative points. If the same herbarium voucher also represents the type material (10^th^ level of value), then it received 70 (7 x 10) cumulative points. In such a case, the highest points (i.e. 70) were taken into account and this specimen was digitised at the first priority, while other specimens with lower points were digitised later, in order of received points.

2. The prioritised specimens were generally checked for preserving condition and presence of the readable labels. They also were preliminarily evaluated to fit the digitisation protocols and available technical facilities at SMNH NASU.

3. The still image of each specimen has been captured using different photosystems available at SMNH NASU. For microslides, the digitisation photo camera Canon EOS 800D (24 Mp) with Canon EF-S 18-55mm f/3.5-5.6 IS STM lens mounted on the horizontal tripod over the light box has been applied. The following presets were set up: ISO 200, f/5.6, exposition 1/250, automatic white balance. Additionally, the camera Olympus DP72 mounted on the trinocular microscope Olympus ВX51 has been used for microphotography purposes. For the digitisation of pinned and fixed specimens, the photo camera Canon EOS 800D (24 Mp) with Canon EF 100mm f/2.8L Macro IS USM lens also mounted on the horizontal tripod over the lightbox has been applied. The images were saved simultaneously in RAW (master file) and JPEG (distributive file) formats in the highest possible resolution. At the moment, the resulting images are stored on the internal SMNH NASU server and only a portion of them has been published online.

4. The data from the labels have been manually filled from the images into Excel tables mapped following DarwinCore standard (TDWG 2024) and separated by taxonomic group.

5. The first step of data quality control has been manually realised by assistants, who checked the initial datasets for typos, technical mistakes and errors.

6. The occurrence records were georeferenced using the data from the field "locality" and OpenStreetMap facilities (OpenStreetMap contributors 2024). The OpenStreetMap has been chosen over other similar web map services as it is well-developed, openly provided under ODbL licence and has extended functionality, allowing checking the elevation. Many toponyms in the OpenStreetMap are provided along with spelling variants and there is an option to add new or correct existing information on the map. This results in better identification of the locality described on the label. The coordinates accuracy has been evaluated in metres and filled in the respective field in the dataset.

7. The second step of data quality control has been realised by collection curators, checking for consistency of provided coordinates and localities descriptions.

8. Separate datasets were merged into the common dataset by the project PI (AN).

9. The third step of data quality control has been realised by the project PI (AN), checking for consistency of provided data in the merged dataset.

10. The dataset has been published using the GBIF IPT (GBIF 2024b).

## Geographic coverage

### Description

**Figure 3.**
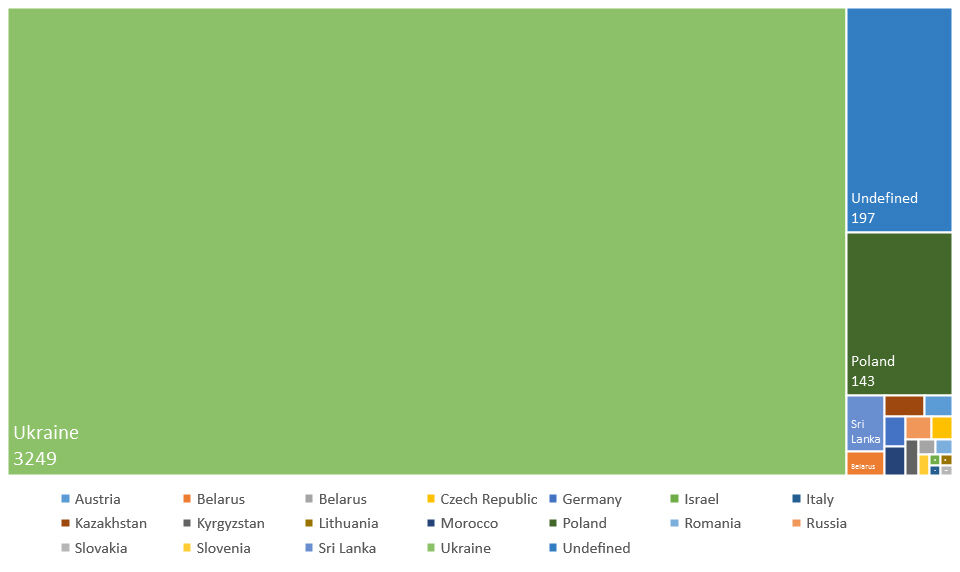
Distribution of the occurrence records in the dataset by countries.

The SMNH NASU mainly hosts natural history collections representing regional flora and fauna. Only certain specimens were collected out of the western part of Ukraine. Therefore, most of the occurrence records provided in the dataset are from Ukraine (3249) and adjacent territories of Poland (143) - Fig. 3. Other currently provided occurrence records cover Sri Lanka (18), Belarus (8), Kazakhstan (7), Austria (5), Germany (5), Morocco (5), the Russian Federation (5) and the Czechia (4), Kyrgyzstan (4), Bosnia and Herzegovina (2), Romania (2), Slovenia (2), Israel (1), Italy (1), Lithuania (1) and Slovakia (1).

## Taxonomic coverage

### Description

**Figure 4.**
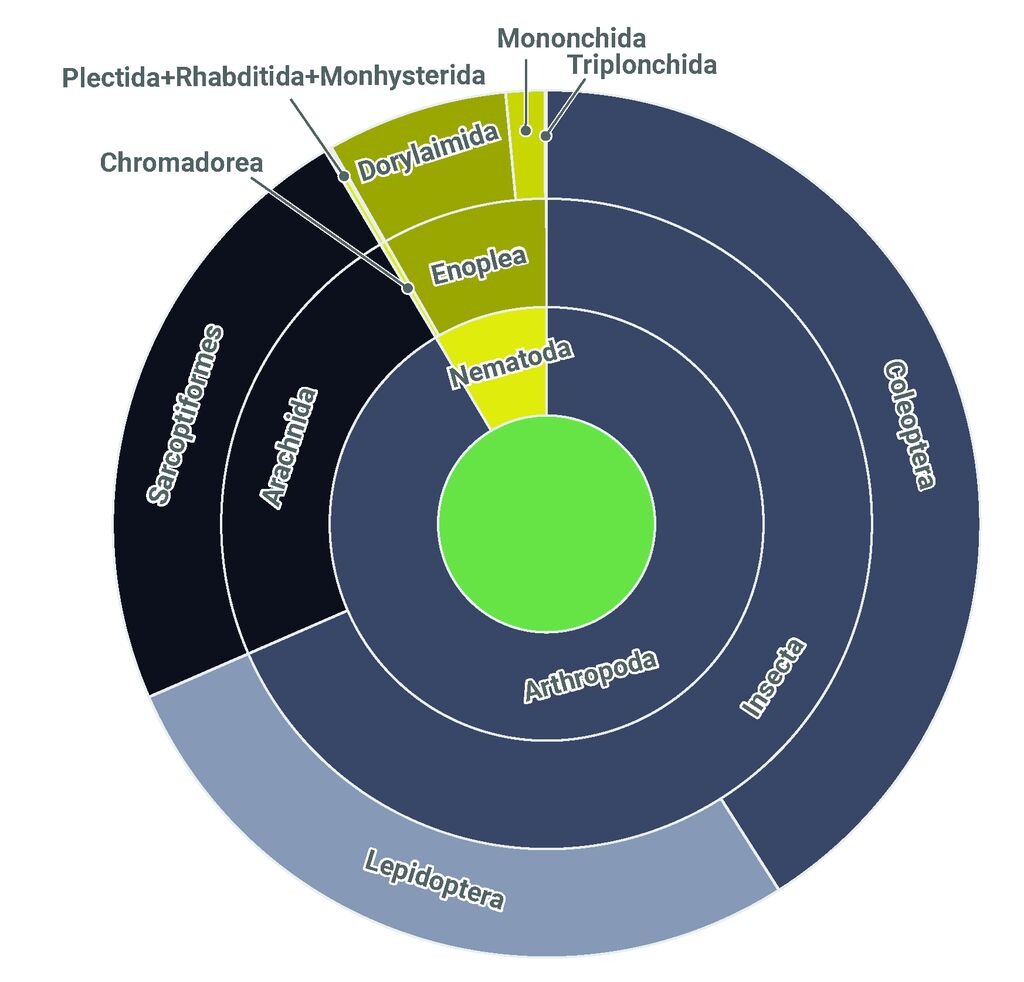
Taxonomic structure of invertebrates represented in the dataset.

The dataset contains occurrence records belonging to two phyla, Arthropoda and Nematoda (Fig. 4 and Table 1).

In the dataset, the phylum Arthropoda is the most diverse both in sense of taxonomy and represented occurrence records (3348 records or 91.5% of the total number of records). It includes holo- and paratypes of *Trechuspseudomontanellus* (35 specimens and respective occurrence records), *Duvaliustranscarpathicus* (2 specimens) and *Duvaliuswerchratskii* (1 specimen). There are also occurrence records about 11 species endemic to the Carpathians (*Duvaliopsispilosella*, *Duvaliuscorpulentus*, *D.roubali*, *D.ruthenus*, *D.subterraneus*, *D.transcarpathicus*, *Trechuscarpaticus*, *T.fontinalis*, *T.latus*, *T.plicatulus* and *T.pseudomontanellus*). Finally, it includes information about the reference collection of oribatid mites typical and the oldest known collection of Lepidoptera for the western part of Ukraine.

The phylum Nematoda is less represented in the dataset by the number of records (312 records or 8.5% of the total number of records). However, the provided data on the phylum Nematoda are extremely important since they represent holo- and paratypes of 15 species described from Ukraine, Poland and Russia – *Anatonchussiddiqii* (5 microscopic slides and respective occurence records), *Clarkuspatricius* (5 slides), *Comiconchuszduni* (2 slides), *Makatinusukrainicus* (5 slides), *Metaporcelaimusconcinnus* (6 slides), *M.declivicaudatus* (5 slides), *M.petrophilus* (4 slides), *Mylonchuluspolitus* Susulovsky, 2000 (4 slides), *Prionchulusfistulosus* (20 slides), *P.hygrophilus* (5 slides), *P.kamchaticus* (2 slides), *P.polonicus* (1 slide), *P.pseudolongus* (2 slides), *Tigronchoidesandrassyi* (2 slides) and *Tridentuluspalustris* (1 slide). Most other microscopic slides of nematodes cited in the dataset, contain the materials of re-descriptions serving as the basis for the contemporary taxonomic understanding of such species as *Acromoldavicusskrjabini* (1 slide), *Longidorusintermedius* (18 slides), *Makatinusaquaticus* (9 slides), *Metaporcelaimusromanicus* (10 slides), *M.ovogranulosus* (7 slides), *Parkellusmenzeli* (3 slides), *P.zschokkei* (3 slides), *Plectusacuminatus* (3 slides) and *P.decens* (5 slides). For six species, i.e. *Longidoruscaespiticola* (22 slides), *L.danuvii* (32 slides), *L.poessneckensis* (12 slides), *Paralongidorusrex* (11 slides), *Prionchulusfistulosus* (20 slides) and *Xiphinemaifacolum* (18 slides), the digitised material includes complete cycles of post-embryonic development, which is important for the taxonomy and phylogeny of nematodes.

## Temporal coverage

### Notes

**Figure 5.**
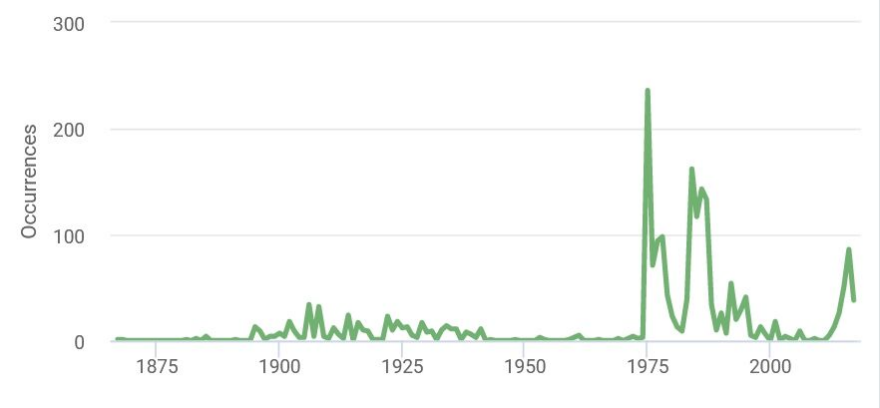
The temporal coverage of the dataset by years.

The general temporal coverage of the dataset is 1867-2017 (Fig. 5). Most of Coleoptera (i.e. Carabidae) specimens were collected in 1975-2006. Most of Lepidoptera specimens were collected in 1903-1940. Most of Sarcoptiformes (i.e. Oribatida) specimens were collected in 1974-1988. Most of Nematoda specimens were collected in 2013-2017.

## Collection data

### Collection name

Invertebrates of SMNH NASU

### Collection identifier

https://ror.org/019qyzj84

### Specimen preservation method

dried, pinned, formalin, alcohol, glycerine, microscopic preparation

### Curatorial unit

188,000 +/- 1,000 specimens

## Usage licence

### Usage licence

Other

### IP rights notes

Creative Commons Attribution License (CC BY 4.0)

## Data resources

### Data package title

SMNH NASU Invertebrates collection

### Resource link

https://doi.org/10.15468/ugtaz7

### Alternative identifiers

https://www.gbif.org/dataset/bdc8cabb-98c5-460b-8173-a7d3fa7b7b19

### Number of data sets

1

### Data set 1.

#### Data set name

SMNH NASU Invertebrates collection

#### Data format

DarwinCore

#### Character set

UTF-8

#### Download URL

https://doi.org/10.15468/ugtaz7

#### Description

The tab-delimited TSV-formatted dataset was created following the DarwinCore standard. It contains 3,660 occurrence records on the digitised specimens of invertebrates deposited in the SMNH NASU (Rizun et al. 2024). This dataset will be dynamically updated with new data along with digitisation and data mobilisation progress.

**Data set 1.** 

Column label	Column description
institutionCode	The acronym in use by the institution having custody of the object(s) or information referred to in the record. In our case, it is SMNH NASU.
institutionID	An identifier for the institution having custody of the object(s) or information referred to in the record. In our case, it is ROR identifier.
basisOfRecord	The specific nature of the data record, for example, preserved specimen or field observation.
occurrenceID	An unique identifier for the Occurrence. In our case, it is UUID ver. 4.
catalogNumber	An identifier for the record within the collection.
verbatimScientificName	A string representing the taxonomic identification as it appeared in the original record.
scientificName	The full scientific name of the taxon including the genus name and specific epithet.
taxonRank	The taxonomic rank of the most specific name in the scientificName.
kingdom	The full scientific name of the kingdom in which taxon is classified.
phylum	The full scientific name of the phylum in which taxon is classified.
class	The full scientific name of the class in which taxon is classified.
order	The full scientific name of the order in which taxon is classified.
family	The full scientific name of the family in which taxon is classified.
genus	The full scientific name of the genus in which taxon is classified.
recordedBy	A person, group or organisation responsible for recording the original Occurrence.
verbatimEventDate	The date of record as it appears in the original publication or specimen label.
eventDate	The date during which an event (e.g. collection of the specimen, photographing of the plant or its registering in the field in any other way), occurred.
countryCode	The standard code (ISO 3166-1-alpha-2) for the country in which the locality occurs.
country	The name of the country in which the locality occurs.
language	The language of data representation for the occurrence record.
locality	The specific description of the place where the specimen was registered or collected.
habitat	The description of the habitat where the specimen was collected or observed.
minimumElevationInMetres	The lower limit of the range of elevation (altitude, usually above sea level), in metres.
maximumElevationInMetres	The upper limit of the range of elevation (altitude, usually above sea level), in metres.
geodeticDatum	The ellipsoid, geodetic datum or spatial reference system (SRS), upon which the geographic coordinates given in decimalLatitude and decimalLongitude are based. In our case, it is WGS84.
decimalLatitude	The geographic latitude (in decimal degrees, using the spatial reference system given in geodeticDatum) of the geographic centre of a locality.
decimalLongitude	The geographic longitude (in decimal degrees, using the spatial reference system given in geodeticDatum) of the geographic centre of a locality.
coordinateUncertaintyInMetres	The horizontal distance (in metres) from the given decimalLatitude and decimalLongitude describing the smallest circle containing the whole of the locality.
identifiedBy	A list of names of people who assigned the taxon to the subject.

## Additional information

### Summary

Despite the minute progress in the digitisation of the SMNH NASU funds (only 11% of invertebrate specimens were digitised and only 2% were published in GBIF), the current activity offers the hope. Over the past five years, there has been an intensification of the digitisation of SMNH NASU collections. If in 2019, the DCBU contained only about 7,000 records on invertebrates, today this database already contains over 20,000 records. Moreover, in 2023, SMNH NASU officially became the GBIF publisher and, since that time, published 12 datasets with ca. 26,000 records. Of this number, about 21,000 records concern herbarium entries and are not limited to collections stored directly in the SMNH NASU (some records concern the information gathered from other herbaria and data parsed from the publications). In this sense, the mobilisation of zoological data lags significantly, which is primarily due to the peculiarities and difficulties of digitising zoological specimens. The lack of experience in data mobilisation and publication in the GBIF also played a significant role in such a lag in the zoological data. However, given the current pace and volume of the SMNH NASU invertebrate collection, in the case of sufficient financial support, it can be fully digitised and presented at the GBIF within the next 10 years.

The current dataset which, at the moment, represents only a small part of the entire SMNH NASU invertebrate collection, will serve as a proxy point to access this virtual collection and will be updated along the digitisation progress. The current digitisation of the SMNH NASU invertebrate collection is focused on the red priority group, limited by the fauna of Ukraine. This group is the most valuable and requires urgent digitisation, since its loss in the case of hostilities will leave an irrevocable legacy. Specimens collected from other countries are out of current digitisation plans and will be digitised only occasionally or on request. The next digitisation round (2025–2030) will involve the specimens belonging to the yellow priority group. The specimens of the green priority group and specimens from othe countries will be digitised in the last digitisation round (2030-2035).

Besides the mentioned plans, we will gladly consider requests for prioritised digitisation from scientists worldwide. We believe that it is most important to digitise those materials that are urgently needed for research purposes. Therefore, please direct your requests to the collection curators, i.e. Coleoptera and other invertebrates - Volodymyr Rizun (novikoffav@gmail.com), Lepidoptera - Kateryna Hushtan (katrinantonyuk@gmail.com), Arachnida - Habriel Hushtan (habrielhushtan@gmail.com) and Nematoda - Andrii Susulovsky (susulovsky@gmail.com).

## Figures and Tables

**Table 1.** The list of invertebrate species and the number of their occurrence records represented in the dataset.

Phylum	Class	Order	Family	Genus	Species	Records
Arthropoda	Arachnida	Sarcoptiformes	Achipteriidae	*Achipteria*	*Achipteriacoleoptrata*	114
Arthropoda	Arachnida	Sarcoptiformes	Achipteriidae	*Parachipteria*	*Parachipteriafanzagoi*	5
Arthropoda	Arachnida	Sarcoptiformes	Achipteriidae	*Parachipteria*	*Parachipteriapunctata*	50
Arthropoda	Arachnida	Sarcoptiformes	Autognetidae	*Autogneta*	*Autognetawillmanni*	6
Arthropoda	Arachnida	Sarcoptiformes	Carabodidae	*Carabodes*	*Carabodeslabyrinthicus*	1
Arthropoda	Arachnida	Sarcoptiformes	Cepheusidae	*Cepheus*	*Cepheuscepheiformis*	3
Arthropoda	Arachnida	Sarcoptiformes	Cepheusidae	*Cepheus*	*Cepheuslatus*	2
Arthropoda	Arachnida	Sarcoptiformes	Cepheusidae	*Reticulocepheus*	*Reticulocepheusgrandis*	2
Arthropoda	Arachnida	Sarcoptiformes	Cepheusidae	*Tritegeus*	*Tritegeusbisulcatus*	2
Arthropoda	Arachnida	Sarcoptiformes	Ceratozetidae	*Ceratozetes*	*Ceratozetesmediocris*	2
Arthropoda	Arachnida	Sarcoptiformes	Ceratozetidae	*Euzetes*	*Euzetesglobulus*	6
Arthropoda	Arachnida	Sarcoptiformes	Ceratozetidae	*Melanozetes*	*Melanozetesmollicomus*	1
Arthropoda	Arachnida	Sarcoptiformes	Ceratozetidae	*Melanozetes*	*Melanozetesmollicomus*	2
Arthropoda	Arachnida	Sarcoptiformes	Chamobatidae	*Chamobates*	*Chamobatesbirulai*	1
Arthropoda	Arachnida	Sarcoptiformes	Chamobatidae	*Chamobates*	*Chamobatesborealis*	2
Arthropoda	Arachnida	Sarcoptiformes	Chamobatidae	*Chamobates*	*Chamobatesvoigtsi*	2
Arthropoda	Arachnida	Sarcoptiformes	Collohmanniidae	*Collohmannia*	*Collohmanniagigantea*	2
Arthropoda	Arachnida	Sarcoptiformes	Crotoniidae	*Camisia*	*Camisiaspinifer*	1
Arthropoda	Arachnida	Sarcoptiformes	Crotoniidae	*Heminothrus*	*Heminothruspeltifer*	55
Arthropoda	Arachnida	Sarcoptiformes	Crotoniidae	*Heminothrus*	*Heminothrustargionii*	4
Arthropoda	Arachnida	Sarcoptiformes	Crotoniidae	*Heminothrus*	*Heminothrusthori*	1
Arthropoda	Arachnida	Sarcoptiformes	Ctenobelbidae	*Ctenobelba*	*Ctenobelbapectinigera*	2
Arthropoda	Arachnida	Sarcoptiformes	Ctenobelbidae	*Ctenobelba*	*Ctenobelbapilosella*	4
Arthropoda	Arachnida	Sarcoptiformes	Damaeidae	*Allobelba*	*Allobelbamacerochaeta*	1
Arthropoda	Arachnida	Sarcoptiformes	Damaeidae	*Belba*	*Belbacorynopus*	4
Arthropoda	Arachnida	Sarcoptiformes	Damaeidae	*Damaeus*	*Damaeusauritus*	8
Arthropoda	Arachnida	Sarcoptiformes	Damaeidae	*Damaeus*	*Damaeusboreus*	3
Arthropoda	Arachnida	Sarcoptiformes	Damaeidae	*Damaeus*	*Damaeusonustus*	1
Arthropoda	Arachnida	Sarcoptiformes	Damaeidae	*Damaeus*	*Damaeusriparius*	12
Arthropoda	Arachnida	Sarcoptiformes	Damaeidae	*Damaeus*	*Damaeussmirnovi*	3
Arthropoda	Arachnida	Sarcoptiformes	Damaeidae	*Metabelba*	*Metabelbapapillipes*	2
Arthropoda	Arachnida	Sarcoptiformes	Damaeidae	*Metabelba*	*Metabelbapulverulenta*	6
Arthropoda	Arachnida	Sarcoptiformes	Damaeidae	*Metabelba*	*Metabelbarohdendorfi*	2
Arthropoda	Arachnida	Sarcoptiformes	Damaeidae	*Subbelba*	*Subbelbapartiocrispa*	2
Arthropoda	Arachnida	Sarcoptiformes	Damaeolidae	*Damaeolus*	*Damaeolusasperatus*	1
Arthropoda	Arachnida	Sarcoptiformes	Eniochthoniidae	*Hypochthoniella*	*Hypochthoniellaminutissima*	1
Arthropoda	Arachnida	Sarcoptiformes	Eremaeidae	*Eremaeus*	*Eremaeushepaticus*	5
Arthropoda	Arachnida	Sarcoptiformes	Eulohmanniidae	*Eulohmannia*	*Eulohmanniaribagai*	3
Arthropoda	Arachnida	Sarcoptiformes	Euphthiracaridae	*Acrotritia*	*Acrotritiaardua*	3
Arthropoda	Arachnida	Sarcoptiformes	Euphthiracaridae	*Acrotritia*	*Acrotritiaduplicata*	1
Arthropoda	Arachnida	Sarcoptiformes	Euphthiracaridae	*Euphthiracarus*	*Euphthiracarusmonodactylus*	6
Arthropoda	Arachnida	Sarcoptiformes	Euphthiracaridae	*Euphthiracarus*	*Euphthiracarusreticulatus*	1
Arthropoda	Arachnida	Sarcoptiformes	Euphthiracaridae	*Microtritia*	*Microtritiaminima*	1
Arthropoda	Arachnida	Sarcoptiformes	Galumnidae	*Allogalumna*	*Allogalumnalongipluma*	2
Arthropoda	Arachnida	Sarcoptiformes	Galumnidae	*Galumna*	*Galumnalanceata*	8
Arthropoda	Arachnida	Sarcoptiformes	Galumnidae	*Pergalumna*	*Pergalumnaaltera*	4
Arthropoda	Arachnida	Sarcoptiformes	Galumnidae	*Pergalumna*	*Pergalumnaformicaria*	1
Arthropoda	Arachnida	Sarcoptiformes	Galumnidae	*Pergalumna*	*Pergalumnamyrmophila*	3
Arthropoda	Arachnida	Sarcoptiformes	Galumnidae	*Pergalumna*	*Pergalumnanervosa*	2
Arthropoda	Arachnida	Sarcoptiformes	Galumnidae	*Pergalumna*	*Pergalumnaobvia*	18
Arthropoda	Arachnida	Sarcoptiformes	Gymnodamaeidae	*Gymnodamaeus*	*Gymnodamaeusbicostatus*	5
Arthropoda	Arachnida	Sarcoptiformes	Hermanniellidae	*Hermanniella*	*Hermannielladolosa*	4
Arthropoda	Arachnida	Sarcoptiformes	Hermanniidae	*Hermannia*	*Hermanniagibba*	1
Arthropoda	Arachnida	Sarcoptiformes	Hypochthoniidae	*Hypochthonius*	*Hypochthoniusluteus*	7
Arthropoda	Arachnida	Sarcoptiformes	Hypochthoniidae	*Hypochthonius*	*Hypochthoniusrufulus*	16
Arthropoda	Arachnida	Sarcoptiformes	Liacaridae	*Adoristes*	*Adoristesovatus*	2
Arthropoda	Arachnida	Sarcoptiformes	Liacaridae	*Liacarus*	*Liacarusbrevilamellatus*	1
Arthropoda	Arachnida	Sarcoptiformes	Liacaridae	*Liacarus*	*Liacaruscoracinus*	4
Arthropoda	Arachnida	Sarcoptiformes	Liacaridae	*Liacarus*	*Liacaruslencoranicus*	1
Arthropoda	Arachnida	Sarcoptiformes	Liacaridae	*Liacarus*	*Liacarusnitens*	1
Arthropoda	Arachnida	Sarcoptiformes	Liacaridae	*Liacarus*	*Liacarussubterraneus*	2
Arthropoda	Arachnida	Sarcoptiformes	Liacaridae	*Liacarus*	*Liacarustubifer*	2
Arthropoda	Arachnida	Sarcoptiformes	Liebstadiidae	*Liebstadia*	*Liebstadialongior*	1
Arthropoda	Arachnida	Sarcoptiformes	Nanhermanniidae	*Nanhermannia*	*Nanhermannianana*	4
Arthropoda	Arachnida	Sarcoptiformes	Neoliodidae	*Poroliodes*	*Poroliodesfarinosus*	2
Arthropoda	Arachnida	Sarcoptiformes	Nothridae	*Nothrus*	*Nothrusanauniensis*	6
Arthropoda	Arachnida	Sarcoptiformes	Nothridae	*Nothrus*	*Nothrusanauniensis*	10
Arthropoda	Arachnida	Sarcoptiformes	Nothridae	*Nothrus*	*Nothruspalustris*	8
Arthropoda	Arachnida	Sarcoptiformes	Nothridae	*Nothrus*	*Nothruspratensis*	1
Arthropoda	Arachnida	Sarcoptiformes	Oppiidae	*Berniniella*	*Berniniellabicarinata*	3
Arthropoda	Arachnida	Sarcoptiformes	Oppiidae	*Dissorhina*	*Dissorhinaornata*	3
Arthropoda	Arachnida	Sarcoptiformes	Oppiidae	*Kulievia*	*Kulieviaparadecipiens*	16
Arthropoda	Arachnida	Sarcoptiformes	Oppiidae	*Multioppia*	*Multioppiaglabra*	8
Arthropoda	Arachnida	Sarcoptiformes	Oppiidae	*Multioppia*	*Multioppialaniseta*	1
Arthropoda	Arachnida	Sarcoptiformes	Oppiidae	*Oppiella*	*Oppiellaneerlandica*	16
Arthropoda	Arachnida	Sarcoptiformes	Oppiidae	*Oppiella*	*Oppiellaneerlandica*	1
Arthropoda	Arachnida	Sarcoptiformes	Oppiidae	*Ramusella*	*Ramusellafurcata*	1
Arthropoda	Arachnida	Sarcoptiformes	Oppiidae	*Rhinoppia*	*Rhinoppiamedia*	6
Arthropoda	Arachnida	Sarcoptiformes	Oppiidae	*Rhinoppia*	*Rhinoppiasubpectinata*	7
Arthropoda	Arachnida	Sarcoptiformes	Oribatulidae	*Oribatula*	*Oribatulatibialis*	1
Arthropoda	Arachnida	Sarcoptiformes	Phenopelopidae	*Eupelops*	*Eupelopsacromios*	4
Arthropoda	Arachnida	Sarcoptiformes	Phenopelopidae	*Eupelops*	*Eupelopscaucasicus*	2
Arthropoda	Arachnida	Sarcoptiformes	Phenopelopidae	*Eupelops*	*Eupelopsoccultus*	5
Arthropoda	Arachnida	Sarcoptiformes	Phenopelopidae	*Eupelops*	*Eupelopsplicatus*	25
Arthropoda	Arachnida	Sarcoptiformes	Phenopelopidae	*Eupelops*	*Eupelopssubuliger*	1
Arthropoda	Arachnida	Sarcoptiformes	Phenopelopidae	*Eupelops*	*Eupelopstorulosus*	18
Arthropoda	Arachnida	Sarcoptiformes	Phenopelopidae	*Peloptulus*	*Peloptulusphaeonotus*	1
Arthropoda	Arachnida	Sarcoptiformes	Phthiracaridae	*Atropacarus*	*Atropacarusphyllophorus*	1
Arthropoda	Arachnida	Sarcoptiformes	Phthiracaridae	*Atropacarus*	*Atropacarusstriculus*	47
Arthropoda	Arachnida	Sarcoptiformes	Phthiracaridae	*Hoplophthiracarus*	*Hoplophthiracarusillinoisensis*	3
Arthropoda	Arachnida	Sarcoptiformes	Phthiracaridae	*Phthiracarus*	*Phthiracarusferrugineus*	7
Arthropoda	Arachnida	Sarcoptiformes	Phthiracaridae	*Phthiracarus*	*Phthiracarusglobosus*	2
Arthropoda	Arachnida	Sarcoptiformes	Phthiracaridae	*Phthiracarus*	*Phthiracaruslaevigatus*	5
Arthropoda	Arachnida	Sarcoptiformes	Phthiracaridae	*Phthiracarus*	*Phthiracaruslaevigatus*	32
Arthropoda	Arachnida	Sarcoptiformes	Phthiracaridae	*Phthiracarus*	*Phthiracaruslentulus*	2
Arthropoda	Arachnida	Sarcoptiformes	Phthiracaridae	*Phthiracarus*	*Phthiracarusligneus*	1
Arthropoda	Arachnida	Sarcoptiformes	Phthiracaridae	*Phthiracarus*	*Phthiracaruslongulus*	64
Arthropoda	Arachnida	Sarcoptiformes	Phthiracaridae	*Phthiracarus*	*Phthiracarusspadix*	18
Arthropoda	Arachnida	Sarcoptiformes	Phthiracaridae	*Steganacarus*	*Steganacarusapplicatus*	4
Arthropoda	Arachnida	Sarcoptiformes	Phthiracaridae	*Steganacarus*	*Steganacaruscarinatus*	32
Arthropoda	Arachnida	Sarcoptiformes	Phthiracaridae	*Steganacarus*	*Steganacarusspinosus*	3
Arthropoda	Arachnida	Sarcoptiformes	Punctoribatidae	*Minunthozetes*	*Minunthozetespseudofusiger*	3
Arthropoda	Arachnida	Sarcoptiformes	Punctoribatidae	*Minunthozetes*	*Minunthozetessemirufus*	10
Arthropoda	Arachnida	Sarcoptiformes	Punctoribatidae	*Punctoribates*	*Punctoribateszachvatkini*	1
Arthropoda	Arachnida	Sarcoptiformes	Quadroppiidae	*Quadroppia*	*Quadroppiaquadricarinata*	1
Arthropoda	Arachnida	Sarcoptiformes	Scheloribatidae	*Euscheloribates*	*Euscheloribatessamsinaki*	1
Arthropoda	Arachnida	Sarcoptiformes	Suctobelbidae	*Kuklosuctobelba*	*Kuklosuctobelbatuberculata*	1
Arthropoda	Arachnida	Sarcoptiformes	Suctobelbidae	*Suctobelba*	*Suctobelbatrigona*	6
Arthropoda	Arachnida	Sarcoptiformes	Suctobelbidae	*Suctobelbella*	*Suctobelbellaalloenasuta*	5
Arthropoda	Arachnida	Sarcoptiformes	Suctobelbidae	*Suctobelbella*	*Suctobelbellapalustris*	2
Arthropoda	Arachnida	Sarcoptiformes	Suctobelbidae	*Suctobelbella*	*Suctobelbellaperpendiculata*	1
Arthropoda	Arachnida	Sarcoptiformes	Suctobelbidae	*Suctobelbella*	*Suctobelbellasimilis*	5
Arthropoda	Arachnida	Sarcoptiformes	Suctobelbidae	*Suctobelbella*	*Suctobelbellasubcornigera*	1
Arthropoda	Arachnida	Sarcoptiformes	Suctobelbidae	*Suctobelbella*	*Suctobelbellasubtrigona*	1
Arthropoda	Arachnida	Sarcoptiformes	Tectocepheidae	*Tectocepheus*	*Tectocepheusvelatus*	1
Arthropoda	Arachnida	Sarcoptiformes	Thyrisomidae	*Banksinoma*	*Banksinomalanceolata*	1
Arthropoda	Arachnida	Sarcoptiformes	Trhypochthoniidae	*Trhypochthonius*	*Trhypochthoniustectorum*	1
Arthropoda	Arachnida	Sarcoptiformes	Xenillidae	*Xenillus*	*Xenillustegeocranus*	1
Arthropoda	Arachnida	Sarcoptiformes	Zetorchestidae	*Zetorchestes*	*Zetorchestesfalzonii*	8
Arthropoda	Insecta	Coleoptera	Carabidae	*Blemus*	*Blemusdiscus*	19
Arthropoda	Insecta	Coleoptera	Carabidae	*Calosoma*	*Calosomaauropunctatum*	4
Arthropoda	Insecta	Coleoptera	Carabidae	*Calosoma*	*Calosomadenticolle*	7
Arthropoda	Insecta	Coleoptera	Carabidae	*Calosoma*	*Calosomaelegans*	3
Arthropoda	Insecta	Coleoptera	Carabidae	*Calosoma*	*Calosomainquisitor*	30
Arthropoda	Insecta	Coleoptera	Carabidae	*Calosoma*	*Calosomainvestigator*	1
Arthropoda	Insecta	Coleoptera	Carabidae	*Calosoma*	*Calosomareticulatum*	1
Arthropoda	Insecta	Coleoptera	Carabidae	*Calosoma*	*Calosomasycophanta*	14
Arthropoda	Insecta	Coleoptera	Carabidae	*Cicindela*	*Cicindelaaltaica*	2
Arthropoda	Insecta	Coleoptera	Carabidae	*Cicindela*	*Cicindelacampestris*	78
Arthropoda	Insecta	Coleoptera	Carabidae	*Cicindela*	*Cicindelacoerulea*	2
Arthropoda	Insecta	Coleoptera	Carabidae	*Cicindela*	*Cicindelagranulata*	4
Arthropoda	Insecta	Coleoptera	Carabidae	*Cicindela*	*Cicindelahybrida*	89
Arthropoda	Insecta	Coleoptera	Carabidae	*Cicindela*	*Cicindelalittoralis*	3
Arthropoda	Insecta	Coleoptera	Carabidae	*Cicindela*	*Cicindelamaritima*	4
Arthropoda	Insecta	Coleoptera	Carabidae	*Cicindela*	*Cicindelanordmanni*	7
Arthropoda	Insecta	Coleoptera	Carabidae	*Cicindela*	*Cicindelasachalinensis*	1
Arthropoda	Insecta	Coleoptera	Carabidae	*Cicindela*	*Cicindelasahlbergii*	2
Arthropoda	Insecta	Coleoptera	Carabidae	*Cicindela*	*Cicindelasoluta*	9
Arthropoda	Insecta	Coleoptera	Carabidae	*Cicindela*	*Cicindelasylvatica*	47
Arthropoda	Insecta	Coleoptera	Carabidae	*Cicindela*	*Cicindelasylvicola*	53
Arthropoda	Insecta	Coleoptera	Carabidae	*Cylindera*	*Cylinderaarenaria*	51
Arthropoda	Insecta	Coleoptera	Carabidae	*Cylindera*	*Cylinderagermanica*	141
Arthropoda	Insecta	Coleoptera	Carabidae	*Duvaliopsis*	*Duvaliopsispilosella*	17
Arthropoda	Insecta	Coleoptera	Carabidae	*Duvalius*	*Duvaliuscorpulentus*	25
Arthropoda	Insecta	Coleoptera	Carabidae	*Duvalius*	*Duvaliusmicrophthalmus*	2
Arthropoda	Insecta	Coleoptera	Carabidae	*Duvalius*	*Duvaliusprocerus*	2
Arthropoda	Insecta	Coleoptera	Carabidae	*Duvalius*	*Duvaliusroubali*	28
Arthropoda	Insecta	Coleoptera	Carabidae	*Duvalius*	*Duvaliusruthenus*	5
Arthropoda	Insecta	Coleoptera	Carabidae	*Duvalius*	*Duvaliussubterraneus*	47
Arthropoda	Insecta	Coleoptera	Carabidae	*Duvalius*	*Duvaliustranscarpathicus*	62
Arthropoda	Insecta	Coleoptera	Carabidae	*Epaphius*	*Epaphiusrivularis*	4
Arthropoda	Insecta	Coleoptera	Carabidae	*Epaphius*	*Epaphiussecalis*	43
Arthropoda	Insecta	Coleoptera	Carabidae	*Lophyra*	*Lophyraflexuosa*	5
Arthropoda	Insecta	Coleoptera	Carabidae	*Omophron*	*Omophronlimbatum*	50
Arthropoda	Insecta	Coleoptera	Carabidae	*Omophron*	*Omophronrotundatum*	2
Arthropoda	Insecta	Coleoptera	Carabidae	*Perileptus*	*Perileptusareolatus*	22
Arthropoda	Insecta	Coleoptera	Carabidae	*Thalassophilus*	*Thalassophiluslongicornis*	10
Arthropoda	Insecta	Coleoptera	Carabidae	*Trechoblemus*	*Trechoblemusmicros*	13
Arthropoda	Insecta	Coleoptera	Carabidae	*Trechus*	*Trechusalpicola*	2
Arthropoda	Insecta	Coleoptera	Carabidae	*Trechus*	*Trechusamplicollis*	15
Arthropoda	Insecta	Coleoptera	Carabidae	*Trechus*	*Trechusaustriacus*	2
Arthropoda	Insecta	Coleoptera	Carabidae	*Trechus*	*Trechuscardioderus*	2
Arthropoda	Insecta	Coleoptera	Carabidae	*Trechus*	*Trechuscarpaticus*	19
Arthropoda	Insecta	Coleoptera	Carabidae	*Trechus*	*Trechusfontinalis*	39
Arthropoda	Insecta	Coleoptera	Carabidae	*Trechus*	*Trechuslatus*	97
Arthropoda	Insecta	Coleoptera	Carabidae	*Trechus*	*Trechusobtusus*	1
Arthropoda	Insecta	Coleoptera	Carabidae	*Trechus*	*Trechusovatus*	3
Arthropoda	Insecta	Coleoptera	Carabidae	*Trechus*	*Trechuspilisensis*	52
Arthropoda	Insecta	Coleoptera	Carabidae	*Trechus*	*Trechusplicatulus*	24
Arthropoda	Insecta	Coleoptera	Carabidae	*Trechus*	*Trechuspseudomontanellus*	35
Arthropoda	Insecta	Coleoptera	Carabidae	*Trechus*	*Trechuspulchellus*	41
Arthropoda	Insecta	Coleoptera	Carabidae	*Trechus*	*Trechuspulpani*	17
Arthropoda	Insecta	Coleoptera	Carabidae	*Trechus*	*Trechusquadristriatus*	101
Arthropoda	Insecta	Coleoptera	Carabidae	*Trechus*	*Trechusrotundipennis*	1
Arthropoda	Insecta	Coleoptera	Carabidae	*Trechus*	*Trechusrubens*	23
Arthropoda	Insecta	Coleoptera	Carabidae	*Trechus*	*Trechussplendens*	5
Arthropoda	Insecta	Coleoptera	Carabidae	*Trechus*	*Trechusstriatulus*	113
Arthropoda	Insecta	Lepidoptera	Brahmaeidae	*Lemonia*	*Lemoniadumi*	12
Arthropoda	Insecta	Lepidoptera	Brahmaeidae	*Lemonia*	*Lemoniataraxaci*	6
Arthropoda	Insecta	Lepidoptera	Drepanidae	*Cilix*	*Cilixglaucata*	12
Arthropoda	Insecta	Lepidoptera	Drepanidae	*Drepana*	*Drepanacurvatula*	8
Arthropoda	Insecta	Lepidoptera	Drepanidae	*Drepana*	*Drepanafalcataria*	18
Arthropoda	Insecta	Lepidoptera	Drepanidae	*Falcaria*	*Falcarialacertinaria*	8
Arthropoda	Insecta	Lepidoptera	Drepanidae	*Sabra*	*Sabraharpagula*	6
Arthropoda	Insecta	Lepidoptera	Drepanidae	*Watsonalla*	*Watsonallabinaria*	6
Arthropoda	Insecta	Lepidoptera	Drepanidae	*Watsonalla*	*Watsonallacultraria*	20
Arthropoda	Insecta	Lepidoptera	Endromidae	*Endromis*	*Endromisversicolora*	28
Arthropoda	Insecta	Lepidoptera	Erebidae	*Arctia*	*Arctiacaja*	26
Arthropoda	Insecta	Lepidoptera	Erebidae	*Callimorpha*	*Callimorphadominula*	12
Arthropoda	Insecta	Lepidoptera	Erebidae	*Catocala*	*Catocalaelecta*	7
Arthropoda	Insecta	Lepidoptera	Erebidae	*Catocala*	*Catocalaelocata*	8
Arthropoda	Insecta	Lepidoptera	Erebidae	*Catocala*	*Catocalafraxini*	6
Arthropoda	Insecta	Lepidoptera	Erebidae	*Catocala*	*Catocalafulminea*	6
Arthropoda	Insecta	Lepidoptera	Erebidae	*Catocala*	*Catocalanupta*	11
Arthropoda	Insecta	Lepidoptera	Erebidae	*Catocala*	*Catocalapromissa*	9
Arthropoda	Insecta	Lepidoptera	Erebidae	*Catocala*	*Catocalasponsa*	17
Arthropoda	Insecta	Lepidoptera	Erebidae	*Coscinia*	*Cosciniacribraria*	2
Arthropoda	Insecta	Lepidoptera	Erebidae	*Coscinia*	*Cosciniastriata*	10
Arthropoda	Insecta	Lepidoptera	Erebidae	*Diacrisia*	*Diacrisiasannio*	15
Arthropoda	Insecta	Lepidoptera	Erebidae	*Diaphora*	*Diaphoramendica*	8
Arthropoda	Insecta	Lepidoptera	Erebidae	*Epatolmis*	*Epatolmisluctifera*	1
Arthropoda	Insecta	Lepidoptera	Erebidae	*Epicallia*	*Epicalliavillica*	15
Arthropoda	Insecta	Lepidoptera	Erebidae	*Eucharia*	*Euchariafestiva*	3
Arthropoda	Insecta	Lepidoptera	Erebidae	*Euplagia*	*Euplagiaquadripunctaria*	10
Arthropoda	Insecta	Lepidoptera	Erebidae	*Hyphoraia*	*Hyphoraiaaulica*	17
Arthropoda	Insecta	Lepidoptera	Erebidae	*Miltochrista*	*Miltochristaminiata*	6
Arthropoda	Insecta	Lepidoptera	Erebidae	*Nudaria*	*Nudariamundana*	1
Arthropoda	Insecta	Lepidoptera	Erebidae	*Parasemia*	*Parasemiaplantaginis*	17
Arthropoda	Insecta	Lepidoptera	Erebidae	*Pericallia*	*Pericalliamatronula*	8
Arthropoda	Insecta	Lepidoptera	Erebidae	*Phragmatobia*	*Phragmatobiafuliginosa*	6
Arthropoda	Insecta	Lepidoptera	Erebidae	*Rhyparia*	*Rhypariapurpurata*	10
Arthropoda	Insecta	Lepidoptera	Erebidae	*Spilarctia*	*Spilarctialutea*	9
Arthropoda	Insecta	Lepidoptera	Erebidae	*Spilosoma*	*Spilosomalubricipeda*	7
Arthropoda	Insecta	Lepidoptera	Erebidae	*Spilosoma*	*Spilosomaurticae*	2
Arthropoda	Insecta	Lepidoptera	Erebidae	*Tyria*	*Tyriajacobaeae*	5
Arthropoda	Insecta	Lepidoptera	Erebidae	*Utetheisa*	*Utetheisapulchella*	1
Arthropoda	Insecta	Lepidoptera	Lasiocampidae	*Cosmotriche*	*Cosmotrichelobulina*	1
Arthropoda	Insecta	Lepidoptera	Lasiocampidae	*Dendrolimus*	*Dendrolimuspini*	12
Arthropoda	Insecta	Lepidoptera	Lasiocampidae	*Euthrix*	*Euthrixpotatoria*	5
Arthropoda	Insecta	Lepidoptera	Lasiocampidae	*Gastropacha*	*Gastropachapopulifolia*	8
Arthropoda	Insecta	Lepidoptera	Lasiocampidae	*Gastropacha*	*Gastropachaquercifolia*	8
Arthropoda	Insecta	Lepidoptera	Lasiocampidae	*Lasiocampa*	*Lasiocampaquercus*	9
Arthropoda	Insecta	Lepidoptera	Lasiocampidae	*Lasiocampa*	*Lasiocampatrifolii*	5
Arthropoda	Insecta	Lepidoptera	Lasiocampidae	*Macrothylacia*	*Macrothylaciarubi*	8
Arthropoda	Insecta	Lepidoptera	Lasiocampidae	*Odonestis*	*Odonestispruni*	14
Arthropoda	Insecta	Lepidoptera	Lasiocampidae	*Phyllodesma*	*Phyllodesmailicifolia*	1
Arthropoda	Insecta	Lepidoptera	Lasiocampidae	*Phyllodesma*	*Phyllodesmatremulifolia*	3
Arthropoda	Insecta	Lepidoptera	Nolidae	*Bena*	*Benabicolorana*	2
Arthropoda	Insecta	Lepidoptera	Nolidae	*Pseudoips*	*Pseudoipsprasinana*	11
Arthropoda	Insecta	Lepidoptera	Nymphalidae	*Aglais*	*Aglaisio*	12
Arthropoda	Insecta	Lepidoptera	Nymphalidae	*Aglais*	*Aglaisurticae*	12
Arthropoda	Insecta	Lepidoptera	Nymphalidae	*Apatura*	*Apaturailia*	44
Arthropoda	Insecta	Lepidoptera	Nymphalidae	*Apatura*	*Apaturairis*	10
Arthropoda	Insecta	Lepidoptera	Nymphalidae	*Araschnia*	*Araschnialevana*	18
Arthropoda	Insecta	Lepidoptera	Nymphalidae	*Euphydryas*	*Euphydryasaurinia*	10
Arthropoda	Insecta	Lepidoptera	Nymphalidae	*Euphydryas*	*Euphydryasmaturna*	5
Arthropoda	Insecta	Lepidoptera	Nymphalidae	*Ladoga*	*Ladogacamilla*	14
Arthropoda	Insecta	Lepidoptera	Nymphalidae	*Limenitis*	*Limenitispopuli*	32
Arthropoda	Insecta	Lepidoptera	Nymphalidae	*Melitaea*	*Melitaeacinxia*	5
Arthropoda	Insecta	Lepidoptera	Nymphalidae	*Melitaea*	*Melitaeadidyma*	19
Arthropoda	Insecta	Lepidoptera	Nymphalidae	*Melitaea*	*Melitaeaphoebe*	6
Arthropoda	Insecta	Lepidoptera	Nymphalidae	*Melitaea*	*Melitaeatrivia*	3
Arthropoda	Insecta	Lepidoptera	Nymphalidae	*Mellicta*	*Mellictaathalia*	20
Arthropoda	Insecta	Lepidoptera	Nymphalidae	*Mellicta*	*Mellictaaurelia*	3
Arthropoda	Insecta	Lepidoptera	Nymphalidae	*Neptis*	*Neptisrivularis*	12
Arthropoda	Insecta	Lepidoptera	Nymphalidae	*Neptis*	*Neptissappho*	16
Arthropoda	Insecta	Lepidoptera	Nymphalidae	*Nymphalis*	*Nymphalisantiopa*	5
Arthropoda	Insecta	Lepidoptera	Nymphalidae	*Nymphalis*	*Nymphalispolychloros*	6
Arthropoda	Insecta	Lepidoptera	Nymphalidae	*Nymphalis*	*Nymphalisxanthomelas*	5
Arthropoda	Insecta	Lepidoptera	Nymphalidae	*Polygonia*	*Polygoniac-album*	12
Arthropoda	Insecta	Lepidoptera	Nymphalidae	*Polygonia*	*Polygoniavaualbum*	1
Arthropoda	Insecta	Lepidoptera	Nymphalidae	*Vanessa*	*Vanessaatalanta*	16
Arthropoda	Insecta	Lepidoptera	Nymphalidae	*Vanessa*	*Vanessacardui*	18
Arthropoda	Insecta	Lepidoptera	Pieridae	*Colias*	*Coliasmyrmidone*	68
Arthropoda	Insecta	Lepidoptera	Pieridae	*Gonepteryx*	*Gonepteryxrhamni*	5
Arthropoda	Insecta	Lepidoptera	Saturniidae	*Aglia*	*Agliatau*	8
Arthropoda	Insecta	Lepidoptera	Saturniidae	*Saturnia*	*Saturniapavonia*	16
Arthropoda	Insecta	Lepidoptera	Saturniidae	*Saturnia*	*Saturniapyri*	12
Arthropoda	Insecta	Lepidoptera	Sphingidae	*Acherontia*	*Acherontiaatropos*	4
Arthropoda	Insecta	Lepidoptera	Sphingidae	*Agrius*	*Agriusconvolvuli*	8
Arthropoda	Insecta	Lepidoptera	Sphingidae	*Deilephila*	*Deilephilaelpenor*	9
Arthropoda	Insecta	Lepidoptera	Sphingidae	*Deilephila*	*Deilephilaporcellus*	5
Arthropoda	Insecta	Lepidoptera	Sphingidae	*Hyles*	*Hyleseuphorbiae*	16
Arthropoda	Insecta	Lepidoptera	Sphingidae	*Hyles*	*Hylesgallii*	6
Arthropoda	Insecta	Lepidoptera	Sphingidae	*Hyles*	*Hyleslineata*	5
Arthropoda	Insecta	Lepidoptera	Sphingidae	*Hyles*	*Hylesvespertilio*	1
Arthropoda	Insecta	Lepidoptera	Sphingidae	*Laothoe*	*Laothoepopuli*	7
Arthropoda	Insecta	Lepidoptera	Sphingidae	*Macroglossum*	*Macroglossumstellatarum*	6
Arthropoda	Insecta	Lepidoptera	Sphingidae	*Mimas*	*Mimastiliae*	9
Arthropoda	Insecta	Lepidoptera	Sphingidae	*Proserpinus*	*Proserpinusproserpina*	2
Arthropoda	Insecta	Lepidoptera	Sphingidae	*Smerinthus*	*Smerinthusplanus*	7
Arthropoda	Insecta	Lepidoptera	Sphingidae	*Sphinx*	*Sphinxligustri*	9
Arthropoda	Insecta	Lepidoptera	Sphingidae	*Sphinx*	*Sphinxpinastri*	8
Arthropoda	Insecta	Lepidoptera	Thyrididae	*Thyris*	*Thyrisfenestrella*	24
Nematoda	Chromadorea	Monhysterida	Monhysteridae	*Tridentula*	*Tridentulapalustris*	1
Nematoda	Chromadorea	Plectida	Plectidae	*Plectus*	*Plectusacuminatus*	3
Nematoda	Chromadorea	Plectida	Plectidae	*Plectus*	*Plectusdecens*	5
Nematoda	Chromadorea	Rhabditida	Cephalobidae	*Acromoldavicus*	*Acromoldavicusskrjabini*	1
Nematoda		Dorylaimida	Aporcelaimidae	*Makatinus*	*Makatinusaquaticus*	9
Nematoda		Dorylaimida	Aporcelaimidae	*Makatinus*	*Makatinusukrainicus*	5
Nematoda		Dorylaimida	Aporcelaimidae	*Metaporcelaimus*	*Metaporcelaimusconcinnus*	6
Nematoda		Dorylaimida	Aporcelaimidae	*Metaporcelaimus*	*Metaporcelaimusdeclivicaudatus*	5
Nematoda		Dorylaimida	Aporcelaimidae	*Metaporcelaimus*	*Metaporcelaimusovogranulosus*	7
Nematoda		Dorylaimida	Aporcelaimidae	*Metaporcelaimus*	*Metaporcelaimuspetrophilus*	4
Nematoda		Dorylaimida	Aporcelaimidae	*Metaporcelaimus*	*Metaporcelaimusromanicus*	10
Nematoda		Dorylaimida	Dorylaimidae	*Crassolabium*	*Crassolabiumneohimalum*	5
Nematoda		Dorylaimida	Longidoridae	*Longidorus*	*Longidorusattenuatus*	15
Nematoda		Dorylaimida	Longidoridae	*Longidorus*	*Longidoruscaespiticola*	22
Nematoda		Dorylaimida	Longidoridae	*Longidorus*	*Longidorusdanuvii*	32
Nematoda		Dorylaimida	Longidoridae	*Longidorus*	*Longidorusdistinctus*	13
Nematoda		Dorylaimida	Longidoridae	*Longidorus*	*Longidoruselongatus*	13
Nematoda		Dorylaimida	Longidoridae	*Longidorus*	*Longidoruseuonymus*	17
Nematoda		Dorylaimida	Longidoridae	*Longidorus*	*Longidorusintermedius*	18
Nematoda		Dorylaimida	Longidoridae	*Longidorus*	*Longidoruspoessneckensis*	12
Nematoda		Dorylaimida	Longidoridae	*Paralongidorus*	*Paralongidorusrex*	12
Nematoda		Dorylaimida	Longidoridae	*Xiphinema*	*Xiphinemaifacolum*	18
Nematoda		Dorylaimida	Longidoridae	*Xiphinema*	*Xiphinemataylori*	6
Nematoda		Dorylaimida	Longidoridae	*Xiphinema*	*Xiphinemavuittenezi*	18
Nematoda		Mononchida	Anatonchidae	*Anatonchus*	*Anatonchussiddiqii*	5
Nematoda		Mononchida	Anatonchidae	*Tigronchoides*	*Tigronchoidesandrassyi*	2
Nematoda		Mononchida	Cobbonchidae	*Comiconchus*	*Comiconchuszduni*	2
Nematoda		Mononchida	Mononchidae	*Clarkus*	*Clarkuspatricius*	5
Nematoda		Mononchida	Mononchidae	*Parkellus*	*Parkellusmenzeli*	2
Nematoda		Mononchida	Mononchidae	*Parkellus*	*Parkelluszschokkei*	3
Nematoda		Mononchida	Mononchidae	*Prionchulus*	*Prionchulusfistulosus*	20
Nematoda		Mononchida	Mononchidae	*Prionchulus*	*Prionchulushygrophilus*	5
Nematoda		Mononchida	Mononchidae	*Prionchulus*	*Prionchuluskamchaticus*	2
Nematoda		Mononchida	Mononchidae	*Prionchulus*	*Prionchuluspolonicus*	1
Nematoda		Mononchida	Mononchidae	*Prionchulus*	*Prionchuluspseudolongus*	2
Nematoda		Mononchida	Mylonchulidae	*Paramylonchulus*	*Paramylonchulusjaponicus*	4
Nematoda		Triplonchida	Tripylidae	*Tripyla*	*Tripylaglomerans*	2
